# A qualitative study of clinician attitudes towards closed‐loop systems in mainstream diabetes care in England

**DOI:** 10.1111/dme.14235

**Published:** 2020-02-04

**Authors:** C. Farrington, H. R. Murphy, R. Hovorka

**Affiliations:** ^1^ THIS Institute University of Cambridge Cambridge UK; ^2^ Wellcome Trust–Medical Research Council Institute of Metabolic Science University of Cambridge Cambridge UK; ^3^ Norwich Medical School University of East Anglia Norwich UK

## Abstract

**Aim:**

Clinicians mediate access to new technologies. Consequently, their views on specific devices may influence user access to diabetes technology in mainstream care. As yet, little is known about clinicians’ views about closed‐loop systems. This qualitative study explored clinicians’ views on the likely impacts of future closed‐loop systems in mainstream diabetes care in England.

**Methods:**

We conducted interviews with 36 clinicians from a range of professional backgrounds in five hospital outpatient clinics (two adult, two pregnancy, one paediatric) in England to explore possible consequences of closed‐loop systems for users and clinicians. Data analysis utilized a framework approach.

**Results:**

Clinicians reported a range of expected benefits for future users, including improved glucose control and quality of life. Expected burdens included continued need for manual input and the risk of losing basic self‐care skills. In terms of future clinical workloads, three clinicians emphasized only positive impacts, seven emphasized both positive and negative impacts, and 17 mentioned only negative impacts. Our most prominent finding, expressed by 24 clinicians, was that closed‐loop systems would generate initial challenges due to the need for staff training, user education and support, and new analytical capacities, alongside existing intra‐clinic variations in technological experience.

**Conclusions:**

Clinicians recognize the value of closed‐loop systems in terms of health benefits, but also identify a range of concerns for both users and healthcare staff, which could impact negatively on user access. Future implementation efforts should address these concerns by providing training and support for healthcare teams, taking varied technological expertise into account.

## What's new?

- Clinician attitudes towards new technologies influence outcomes in mainstream care. Little is known about clinician views about the likely impacts of future closed‐loop systems in diabetes care.
- Alongside benefits and burdens for users, clinicians expect closed‐loop systems to generate health service challenges due to heightened needs for training, user support and analytical capacities.
- Clinicians identify a range of concerns for both users and staff, which could impact negatively on user access. Future implementation efforts should address these concerns by providing training and support for healthcare teams.

**What's new?**

- Clinician attitudes towards new technologies influence outcomes in mainstream care. Little is known about clinician views about the likely impacts of future closed‐loop systems in diabetes care.
- Alongside benefits and burdens for users, clinicians expect closed‐loop systems to generate health service challenges due to heightened needs for training, user support and analytical capacities.
- Clinicians identify a range of concerns for both users and staff, which could impact negatively on user access. Future implementation efforts should address these concerns by providing training and support for healthcare teams.

## Introduction

In the absence of clinically available bioengineered solutions such as encapsulated islet cells, the most promising therapeutic option for people with type 1 diabetes is the use of closed‐loop systems 1, 2. Closed‐loop systems use algorithms to process continuous glucose monitoring (CGM) data and deliver precise and frequently updated doses of fast‐acting insulin to users via wearable insulin pumps. Studies of closed‐loop systems demonstrate significantly improved glycaemic control for a wide range of users, with associated psychosocial benefits including perceived freedom from diabetes, peace of mind and improved sleep 2, 3, 4. From a health systems perspective, widespread use of closed‐loop systems could reduce long‐term complications and associated healthcare spending 5. One US‐focused analysis suggested that closed‐loop technology, although requiring substantial initial investment, could lead to cumulative Medicare savings of US $937 million after 25 years 6. Alongside these promising findings, psychosocial research has identified a number of user experience challenges that could limit long‐term usage in mainstream care, including limited trust in automated insulin delivery, ‘deskilling’ (loss of basic diabetes self‐care capacities) and increased time spent thinking about diabetes 2, 3, 4, 7.

To date, less attention has been paid to clinicians’ views on closed‐loop systems, despite their crucial role in mediating access to diabetes technology and related support 8. In the vocabulary of candidacy theory, clinicians engage in negotiations with potential users, or ‘candidates’, in order to adjudicate whether they meet relevant access criteria 9. Yet different clinicians may interpret access criteria in different ways, potentially leading to variable access to treatment (as demonstrated previously in a wide range of clinical settings) 10. In particular, clinicians may hold varied opinions regarding the clinical and psychosocial benefit of new technologies and/or their likely impact on service delivery 11. These views could decrease their willingness to issue positive adjudications when these are otherwise warranted, due to fears regarding user burdens and/or increased clinician workload. It is possible that varied clinician attitudes towards technology may have played a role in comparatively low and geographically uneven levels of access to existing diabetes technologies such as pumps and CGM in England, despite nationwide guidelines issued by the National Institute for Health and Care Excellence (NICE) 8, 12.

NICE guidelines for future closed‐loop systems have yet to be formulated. Nevertheless, it is probable that future access arrangements will continue to require clinician adjudications regarding individual candidate users. Users’ access to future closed‐loop systems may therefore be influenced by varied clinician viewpoints relating to new technology in general and/or closed‐loop systems in particular. Although some studies have explored clinician views about current diabetes technologies such as pumps and CGM 13, 14, 15, 16, 17, clinician perspectives on closed‐loop systems remain poorly understood. To explore clinician views on closed‐loop systems and their potential impacts on future mainstream care for people with type 1 diabetes in England, we aimed to investigate two key areas where clinician attitudes might be especially relevant to future adoption: (1) clinicians’ expectations regarding the likely mix of benefits and burdens experienced by future users, and (2) the potential impact of closed‐loop system on future clinical workloads.

## Participants and methods

### Design and setting

This was a qualitative study using semi‐structured interviews. We carried out 36 interviews with clinicians working at five hospital diabetes outpatient clinics serving adult (two clinics), pregnant (two clinics) and paediatric (one clinic) populations with type 1 diabetes in three hospitals in England, chosen to provide a range of geographical, socio‐economic and technology use contexts. Hospital 1 is a large teaching hospital in an affluent area in the east of England, with high levels of technology use; hospital 2 is a teaching hospital in a less affluent area, also situated in the east of England, with lower levels of technology use; and hospital 3 is a large teaching hospital in the north‐west of England, also in a less affluent area, and with low levels of technology use.

### Participant recruitment

Following ethics approval from the Health Research Authority (HRA; reference 18/HRA/115), we received permission from local National Health Service (NHS) trusts to approach members of outpatient clinic staff for interview. We aimed to sample a range of professions in each clinic, including dieticians, obstetricians, anaesthetists, midwives and psychologists, alongside nurses and physicians. We contacted potential participants via email, with a participant information sheet and consent form, and offered all participants an interview in person or by telephone at a convenient time, date and place. In addition, we used a snowball sampling approach to identify additional staff for interview, asking participants for recommendations of other suitable candidates. We attempted to interview clinicians from a range of professional backgrounds (see Table 1 for participant characteristics) and with varied familiarity with closed‐loop technologies, ranging from extensive personal involvement in trials (*n* = 10) to clinicians with very limited knowledge (*n* = 6). The remaining clinicians (*n* = 20) evidenced some familiarity with closed‐loop systems, often derived from media reports or published papers. For participants who were unfamiliar with closed‐loop systems, CF gave brief descriptions of closed‐loop technology. Our study complied with European Medicines Agency Guidelines for Good Clinical Practice.

**Table 1:** Participant characteristics

Location	Outpatient clinic population	Professional background							
		General medicine/endocrinology (Phys)	Nursing (Nurs)	Dietetics (Diet)	Obstetrics (Obs)	Midwifery (Midw)	Anaesthesiology (Anaest)	Psychology (Psych)	Total
Hospital 1	Pregnancy (PR)	1	2	2	2	1	–	–	8
	Paediatric (PA)	6	2	3	–	–	–	1	12
Hospital 2	Pregnancy (PR)	2	3	1	1	1	1	–	9
	Adult (AD)	4	–	–	–	–	–	–	4
Hospital 3	Adult (AD)	2	1	–	–	–	–	–	3
Total		15	8	6	3	2	1	1	36

Participants are identified using the following naming convention: hospital number/clinic population/profession/number of interviewee within clinic. Abbreviations for clinic population and profession are given in parenthesis in relevant headings of Table 1.

### Data collection

CF conducted interviews in person (*n* = 29) and by telephone (*n* = 7), between October 2017 and June 2018. All participants gave informed consent to participate and to allow digital recording and transcription of interviews. We used a semi‐structured topic guide informed by relevant literature and designed to allow for the exploration of a range of issues, including clinician views about future closed‐loop usage, existing diabetes technologies and organizational culture in outpatient clinics. Our topic guide focused on four key topic areas: knowledge of closed‐loop systems, user experience of closed‐loop technology, the likely impact of closed‐loop technologies on user access to technology, and possible implications for future clinical workload (see Doc. S1 for detailed topic guide). Interviews lasted between 28 and 73 min, with an average time of 47.5 min, a median time of 48 min and an interquartile range of 11 min.

## Data analysis

We analysed the data using a combination of thematic and framework analysis approaches, informed by theories of sense‐making, according to which attitudes towards technology are influenced by preceding experiences, attitudes and values in conjunction with the ‘affordances’, or capacities, of specific devices or systems^18^. Initial coding of interview transcripts took place alongside data collection to identify key themes and generate a provisional coding structure. We then utilized this provisional structure to undertake an initial thematic analysis, using QSR NVivo software (see Table S1 for details of coding structure). Our thematic analysis approach used a six‐stage approach: familiarization with the data, generating initial codes, searching for themes, reviewing themes, defining and naming themes, and producing an overall analysis 19.

We then supplemented our thematic analysis with framework analysis, involving the use of a matrix with cells into which summary qualitative data are entered by category (rows) and cases (columns) 20. This allowed us to identify and explore patterns (categories) that cut across individual clinician attitudes (cases). We focused in particular on two key areas: clinician attitudes to envisaged benefits and burdens for users using closed‐loop technologies, and beliefs regarding the impact of future closed‐loop systems on clinical workloads. We also used the matrix to record clinicians’ professional background, clinic location and antecedent knowledge of closed‐loop technologies and/or trials.

## Results

Despite varied professional backgrounds (and varied levels of involvement in mediating technology access), varied degree of familiarity with closed‐loop technology and varied clinic characteristics (including location, population and levels of technology use), our analysis found broad agreement between clinicians across two key thematic areas relevant to future closed‐loop usage in England: (1) envisaged benefits and burdens for users; and (2) the potential impact of closed‐loop technologies on future clinical workloads.

### Users and closed‐loop systems: expected benefits and burdens

More than half of participants (*n* = 22) expected both benefits and burdens to arise from future use of closed‐loop by users. Of the remaining interviewees, four clinicians mentioned only burdens; one mentioned only benefits; and six mentioned neither benefits nor burdens due to limited knowledge of closed‐loop systems. In the following sections, envisaged benefits and burdens are separated for clarity, although these were often mentioned side‐by‐side by interviewees, reflecting the complex realities of clinical practice.

#### Envisaged benefits of closed‐loop systems for users

A number of clinicians saw closed‐loop systems as the next step in insulin delivery technologies. One physician referred to closed‐loop as ‘the gold standard in terms of insulin management’ (2/AD/Phys/1), whereas others described it as ‘revolutionary’ (1/PA/Diet/9) and ‘the way forward’ for diabetes care (2/PR/Nurs/7). Several emphasized the improved glycaemic control offered by closed‐loop systems:

When you're targeting that HbA_1c_ … overnight blood sugars make a big difference, post‐meal blood sugars make a big difference and … we know closed‐loop can really help to achieve that … [T]hat's really challenging to achieve on a pump or MDI [multiple daily injections]. (1/PR/Diet/2)

In addition to highlighting improved control for users in general, some interviewees anticipated particular benefit for users with lower levels of engagement in self‐care: ‘[It's] particularly good for patients who aren't very motivated … because the difficult stuff will be done for them’ (2/PR/Obs/1). Some clinicians also emphasized potential improvements in quality of life arising from the delegation of self‐care to closed‐loop systems. Specific benefits in this context included improved sleep, reduced diabetes burnout and a sense of freedom from self‐care burdens: ‘in the long run, [closed‐loop will] give [users] a bit of freedom, that they haven't had for all these years, where they've just been looking at diabetes numbers’ (1/PA/Diet/10).

#### Envisaged burdens of closed‐loop systems for users

Although most clinicians recognized at least some potential benefits of closed‐loop usage, they also highlighted many potential burdens that users of closed‐loop systems might experience in mainstream care. Some related to technical and logistical challenges such as the need for users to be permanently attached to devices and to carry support equipment with them. Other concerns centred on what were perceived to be overly cautious (and non‐user‐modifiable) control algorithms, and on the continued need for human input. As one nurse stated, closed‐loop systems are still ‘hybrid’ systems, meaning that users ‘still have to carb‐count and put those things in’ to activate manual prandial bolusing, as well as undertaking frequent testing and calibrating (1/PA/Nurs/7). One physician added that if future closed‐loop systems required only ‘minimum’ human input it would be ‘wonderful, but we're not there yet’ (2/AD/Phys/2).

In addition to these technical burdens, clinicians raised two broad areas of concern: the risk of deskilling and the challenge of surrendering self‐care control to algorithmic closed‐loop systems. With regard to deskilling, one physician expressed their concern that closed‐loop users might lose familiarity with more basic self‐care skills required for multiple daily injections:

If something does go pear‐shaped, they've got to make decisions, they've got to revert perhaps to older technology or to no technology … [Does] using closed‐loop mean that patients and families will deskill themselves … and if things go wrong, they don't know what to do[?] (1/PA/Phys/8)

Another physician stated that closed‐loop users ‘still need to be able to know how to manage hypoglycaemia, they still need to know how to manage ketones … because [closed‐loop] doesn't answer all those problems’ (2/AD/Phys/3).

Challenges of surrendering control, secondly, arise because closed‐loop systems partially eliminate self‐care tasks of monitoring and responding to blood glucose levels. Although this is, in part, an empowering feature of this technology, it also requires people with diabetes to delegate (frequently long‐standing) self‐care routines to an automated system. In this context, several interviewees cited users’ long experience of self‐care as a complicating factor in closed‐loop system adoption. One nurse described the challenge as follows:

[P]eople are going to need a lot of reassurance … They've got to step back, haven't they, [from] all the work they've been doing and the psychological control they've had, because … you need to have a bit of OCD in order to go on a pump and have good diabetes control. So then all of a sudden they're told to not do that anymore and leave it to the closed loop system to do it. (3/AD/Nurs/2)

In this context, one physician suggested that clinicians tend to underestimate the anxiety that new technologies such as closed‐loop systems can cause for users, which ‘can be very, very disabling to diabetes [self‐]care’ (3/AD/Phys/1). A number of clinicians also highlighted the difficulty of predicting users’ acceptance of, or resistance to, closed‐loop usage, describing this as ‘the great unknown’ (3/AD/Phys/3) and as a potential barrier to closed‐loop success.

### Closed‐loop technology and clinical workload

The prevailing view in the context of future clinical workloads was that closed‐loop systems would generate additional short‐ to medium‐term challenges due to the need for staff training, user education and support, and new analytical capacities. Of the 27 clinicians who expressed views regarding future workload, three mentioned only positive impacts, seven mentioned both positive and negative impacts, and 17 mentioned only negative impacts.

#### Positive envisaged impacts on clinical workload

Three clinicians suggested that closed‐loop systems should ‘theoretically … free up time’ (1/PR/Obs/6) because algorithmic insulin delivery would allow users to achieve improved control with less need for intensive clinical input: ‘it will certainly reduce the workload of … medical teams in terms of managing diabetes and the outcome will be spectacularly better for patients’ (1/PR/Nurs/7). Others raised the possibility that the improved glycaemic control offered by closed‐loop systems might help users to avoid future complications, which in turn would reduce the clinical effort needed to treat them. One midwife framed this possibility in terms of healthcare spending, suggesting that ‘when you think of the complications, it would be a lot cheaper to [use closed‐loop systems than not]’ (2/PR/Midw/7).

Several clinicians focused on the increased availability of data arising from system readouts, which some saw as reducing future workloads by limiting the need for face‐to‐face contact. One physician suggested that this could reduce workload since users ‘can [upload] data … and they can send it in’ (2/PR/Phys/2). Others pointed instead to the potential for more efficient (rather than more remote) clinical work, since more user data means

there will be … no second‐guessing about what's going on … in‐between [clinical encounters] … [W]e will have a picture, if you like, of everything that's going on. (2/PR/Phys/8)

In the context of highly variable geographical availability of diabetes outpatient clinics with the capacity to support diabetes technology usage, one physician interpreted future closed‐loop systems as ‘democratizing’ access to diabetes technology use by allowing a wider range of clinicians to supervise the use of new systems: ‘[C]losed loop should make pump therapy much, much more straightforward … and that would take away, I think, some of the disparity in access to skilled teams’ (2/PR/Phys/4). Others suggested that mainstream closed‐loop systems could reduce variations in terms of care delivery within clinics, because the advanced capacities offered by closed‐loop technology may reduce the need for advanced technological knowledge on the part of different clinic personnel: ‘[S]ome of the variability would be taken out of what we're offering’ (1/PA/Diet/10).

#### Negative envisaged impacts on clinical workload

Although some clinicians identified potentially positive impacts of closed‐loop systems on future workload, most were more negative. Participants highlighted three main potential challenges: additional user training needs, time pressure in consultations from increased data analysis requirements, and risks of decreasing user engagement over time.

First, several participants anticipated the need to provide additional training and education to help users cope with the logistical demands of using and maintaining hybrid closed‐loop systems, as well as the initial emotional challenge of surrendering control to an algorithm. One physician stated, for example, that new users ‘will need constant guidance … on how to manage [closed‐loop systems] on a day‐to‐day basis’ (3/AD/Phys/1). Clinicians expected particularly high demands for guidance at the start of closed‐loop usage, and for older users: ‘[I]n the initial stages … I think there will be a lot of hand‐holding. [T]he people who are bit older … will probably be the ones who are ringing us constantly’ (3/AD/Nurs/2).

Some concerns focused, secondly, on the additional data analysis requirements arising from closed‐loop system usage, which were seen as challenging in terms of constrained consultation timeframes:

I think what it would do is probably increase the amount of time spent looking at glucose readings … [In t]he current model in the clinic you … spend all of maybe 20 or 30 seconds looking at their blood glucose concentrations … Now when you are then presented with, potentially, pages and pages of output it may take a lot longer to analyse that. (2/AD/Nurs/2)

Third, there were concerns that users of closed‐loop systems in home‐living conditions may gradually exhibit suboptimal behaviour and technology use, potentially leading to additional clinical work arising from poorer control. In contrast to the initial hurdle of surrendering control over self‐care, they suggested that the challenge presented by declining engagement was likely to increase over time. One dietician invoked her experience of previous closed‐loop trials to describe how trial participants became ‘a bit more lax … like they thought, oh, it'll be alright because closed‐loop will pick it up’ (1/PA/Diet/10). In extremis, one obstetrician expressed concern that some users might not ‘take any notice’ of their system ‘going completely wrong’ (1/PR/Obs/6). Clinicians suggested that the same technological capacity that allows a ‘broader range of people to [attain] an acceptable level of control’ (2/PR/Phys/4) also risks encouraging the idea that closed‐loop technology is ‘going to do everything for [users, and] … fix everything’ (1/PR/Nurs/5).

In addition to highlighting specific challenges, clinicians also identified hurdles in terms of meeting these challenges, reflecting wider concerns regarding current pressures on clinicians working with people with type 1 diabetes 11. In terms of providing additional training, for example, several clinicians suggested that providing support for closed‐loop usage is ‘a big ask’, because many clinicians at present ‘don't [even] know anything about … pump[s]’ and since NHS resources are ‘already so stressed’ (2/AD/Nurs/1). Counterbalancing some participants’ optimism regarding the ‘democratization’ of care delivery within and across clinics, a number of interviewees were troubled by the range of preparedness and technological capacity evidenced by different clinics, with implications for their ability to meet challenges posed by closed‐loop adoption. One physician stated that ‘some centres who are very well‐funded will take up technology quicker than others purely because they can afford to hire … experienced staff … who have some knowledge’ (3/AD/Phys/1). Referring to his own clinic, one physician said: ‘I don't think we are prepared enough … [or] have enough resources to be able to provide it’ (2/AD/Nurs/3).

## Discussion

Participants in this study expected that the introduction of closed‐loop technology into mainstream care in England would generate challenges as well as benefits for both users and clinicians, with potential ramifications for clinician adjudications regarding user candidacy for access to this technology in future. Participants acknowledged the potential of closed‐loop systems in terms of user well‐being and clinical effectiveness, but tended to emphasize pessimistic accounts of technology use in day‐to‐day self‐care and clinical oversight, especially (but not only) with regard to the period immediately following technology adoption. Participants offset envisaged benefits for people with type 1 diabetes (e.g. improved glycaemic control and quality of life) by emphasizing potential burdens arising from the difficulty of surrendering control, the continued need for human input, and the risk of losing basic self‐care capacities. Clinicians similarly identified a range of potential benefits arising from closed‐loop usage, including lower levels of clinical input in day‐to‐day diabetes care, fewer long‐term complications for people with diabetes, and more efficient care arising from greater availability of glucose data. However, they also highlighted a range of potential challenges, including the need to provide additional technology‐related training for users, spend more time interpreting user data in consultations, and deal with complications arising from disengaged users. They also emphasized the difficulty of meeting these challenges in the wider NHS context, characterized by underfunded and overstretched services and high variability in inter‐ and intra‐clinic technical capacity.

As described in candidacy theory approaches to health service access, clinicians are required to mediate access to services, including technology and associated support services, by reaching adjudications regarding whether potential users – ‘candidates’ – meet relevant access criteria 9. Our findings suggest that clinicians’ adjudications regarding closed‐loop usage may be impacted by concerns regarding both user burdens and implications of closed‐loop technology for clinical workload. If eligible users are prevented by clinicians from gaining access to new technologies because of fears regarding user experience and health service factors, the undoubted benefits of closed‐loop technologies may not be fully realized at the population level. To forestall this eventuality, clinicians involved in the use of closed‐loop systems are themselves likely to need significant additional support to introduce and support the technology in routine care, not least because psychosocial research suggests that some users could rule themselves out of candidacy for long‐term use 7.

Our participants did not always agree with each other. Some clinicians suggested, for instance, that the automated character of closed‐loop technology could ‘democratize’ technology access by reducing variations in care provision, whereas others argued that the mainstream adoption of closed‐loop could be challenged by inter‐clinic variations in technological expertise. As such, our study raises, but does not settle, the question of whether future closed‐loop systems should be provided by a small number of specialist centres, potentially improving care quality at the expense of user ease of access, or by a larger number of geographically dispersed outpatient clinics, which would increase ease of access but risk introducing variations in the quality of care provision. Our participants’ views also differed in some ways from findings of previous user‐focused studies, which emphasized a mix of benefits and burdens for users of closed‐loop systems 2, 3, 4. For example, although users in one study believed their need for clinical support would decline following an initial familiarization period 21, clinicians in our study voiced concerns regarding the need for ongoing and possibly increased support for users over time, as a result of factors such as increased data analysis requirements and declining user engagement.

Strengths of our study include in‐depth interviews with clinicians from different backgrounds serving a range of populations, and with varied experience of closed‐loop technology. Our study is limited by uneven numbers of interviewees serving pregnant (*n* = 17), paediatric (*n* = 12) and adult (*n* = 7) populations, and by the dominance of physicians (*n* = 15) as opposed to professions such as nursing (*n* = 8) and dietetics (*n* = 6). Self‐selection bias is possible insofar as clinicians who agreed to participate may have been positively disposed towards closed‐loop technology and/or participation in research projects. The geographical spread of participants was limited by the low number of clinicians recruited at hospital 3 (*n* = 3), and because a second clinic at hospital 3, serving the paediatric population, declined to participate. Although we aimed to recruit the widest possible range of participants, it is possible that clinicians working in other contexts may have different views regarding the introduction of closed‐loop technologies into mainstream care. Future research could investigate the views of clinicians working in a wider variety of geographical settings (including settings beyond the UK), and serving a wider range of populations, including minority ethnic populations whose cultural beliefs may present challenges for closed‐loop adoption 15. Future work could also explore the implications of users creating and using their own closed‐loop systems, bypassing health service access arrangements and potentially requiring distinctive support arrangements 22.

In conclusion, our findings suggest that the introduction of closed‐loop systems to mainstream care in England may create new challenges as well as benefits, for both users and clinicians. In order to minimize challenges and maximize benefits, implementation processes should take account of clinician concerns and ensure the provision of adequate training and staffing resources. Key user‐focused training needs for clinicians involved in closed‐loop care will include technical support, data analytics and aspects of user experience associated with use of automated insulin therapy, ranging from the need to surrender control to the need to maintain basic self‐care capacities. Ideally, additional resources should be targeted to reduce intra‐clinic variation in technological expertise, especially if closed‐loop care is to be provided in non‐specialist clinics.

## Funding sources

CF is supported by a JDRF Strategic Research Award (2‐SRA‐2017‐359) and by the Health Foundation's grant to the University of Cambridge for the Healthcare Improvement Studies (THIS) Institute. HRM conducts independent research supported by the National Institute for Health Research (NIHR Career Development Fellowship, CDF‐2013‐06‐035), and is supported by Tommy's charity. RH is supported by NIHR Cambridge Biomedical Research Centre, JDRF, European Union Horizon 2020 research and innovation programme (grant agreement no 731560), The Leona M. and Harry B. Helmsley Charitable Trust, Efficacy and Mechanism Evaluation Programme NIHR (14/23/09), and Wellcome Trust Strategic Award (100574/Z/12/Z).

## Competing interests

CF has no conflicts of interest. HRM has received honoraria for speaking engagements from Medtronic, Roche, Novo Nordisk, and Eli‐Lilly, and is a member of the Medtronic European Advisory Board. RH has received speaker honoraria from Eli Lilly and Novo Nordisk, serving on advisory panel for Eli Lilly and Novo Nordisk, receiving license fees from B. Braun and Medtronic, having served as a consultant to B. Braun, and patents and patent applications related to closed‐loop.

## Author contributions

CF, HM and RH conceived and planned the study. CF conducted and analysed the interviews, and drafted the manuscript. HM and RH provided feedback on the manuscript. All authors approved the final version for submission. CF is responsible for the content of the manuscript.

## Supporting information


**Doc S1.** Interview topic guide.
**Table S1.** Thematic analysis coding framework.
