# Supplementary material for: A qualitative study of clinician attitudes towards closed‐loop systems in mainstream diabetes care in England
Source: Diabet Med. 2020 Feb 4;37(6):1023–9. doi: 10.1111/dme.14235 (PMC7317734; doi:10.1111/dme.14235)
Supplement: Supplementary file 1 — Doc S1. Interview topic guide. Table S1. Thematic analysis coding framework. [file DME-37-1023-s001.docx]

Supporting Information

**Doc. S1. Interview Topic Guide**

[To be read to interviewees:] The interview has two main parts, focusing on:

- Your attitudes to, and experience of, diabetes technology as used by patients; and
- The organisational and professional culture at your clinic

STRUCTURE:

**Section 1: Diabetes Technology**

1. Attitudes to technology in general
2. Attitudes to non-closed-loop technologies
3. Attitudes to closed-loop technologies

**Section 2: Clinic Culture**

1. Organisational culture
2. Multi-disciplinary working and team climate
3. Resources

QUESTIONS:

**Section 1: Diabetes Technology**

1. Attitudes to technology in general
2. How would you describe your attitude towards new technology in general, i.e. outside work? (Prompt: would you describe yourself as an early adopter?)
3. Have you ever used wearable technologies such as activity trackers?
4. Do you think technology in general makes work easier or more difficult?

Moving on to your clinical experience of diabetes technologies like insulin pumps and CGM:

1. Attitudes to non-closed-loop technologies
2. Roughly what proportion of your patients currently use insulin pumps and CGM sensors?
   1. In what ways have you been involved with these patients in terms of prescribing technology, training processes, advising and/or troubleshooting?
   2. If so, what kind of training have you received for this work?
   3. How did you find the process of learning your way around these technologies?
3. Are requests for new technologies usually patient-led, clinician-led, or a mix?
   1. When patient-led: do particular kinds of patient tend to ask for pumps? If so, do they tend to give similar reasons, or do they vary between patients?
   2. When clinician-led: what prompts you or your colleagues to recommend technologies to patients? What role does NICE guidance play in such recommendations?
   3. Either way: are there sometimes differences between clinician and patient views regarding candidacy? If so, how are these resolved?
4. What is the process – and how formalised is it – for considering a patient’s candidacy for (e.g.) a pump?
   1. Is there a dedicated pathway for access to pumps/CGM? If so, could you describe it?
   2. Are there specific meetings to refer patients to pathways?
   3. What information is brought to bear in candidacy discussions – past experience, specialist knowledge, biomedical data, patient records, personality, funding situation?
   4. Do patients ever drop out in the middle of the process? If so, why?
   5. Are you or your colleagues concerned about the risk of a two-tier population arising, with a divide between those who can access technology and those who can’t?
   6. What role does NICE guidance play in the process?
   7. Do you think NICE guidance is open to interpretation? How would you define, for example, “disabling hypoglycaemia”?
5. Do you think it’s possible to predict accurately which patients will benefit from diabetes technologies?
   1. If so: what kinds of information are most relevant?
6. When patients start using technologies that are new to them:
   1. What kind of expectation management is necessary?
   2. Do you think patients are able to change the way they interact with technology?
7. Attitudes to closed-loop technologies
8. Have you ever been involved in closed-loop trials?
   1. If yes: which studies? How did this experience affect your understanding of, and attitude towards, closed-loop systems?
   2. If not: can you tell me what you know about closed-loop systems, and how you came across your knowledge (e.g. research papers, blogs, colleagues)?
9. Do you think you would see a similar range of success for patients using closed-loop systems as you would for (e.g.) insulin pumps?
   1. How might patient experience differ as a result of closed-loop technology, as opposed to insulin pumps and CGM?)
10. Do you think different discussions would take place in the clinic regarding which patients to recommend for use of closed-loop systems, as opposed to existing technologies?
    1. If so: which new factors in particular will need to be taken into account?
11. How confident would you be in terms of advising patients how to use these systems?
    1. What kind of additional training might be required?
    2. Would you be more concerned about patients using these systems unsupervised than you are about existing technologies?
    3. Are there any particular challenges that might emerge with the specific population your clinic serves, or in your geographical area?
12. Do you think these systems would require the provision of additional support systems, compared to existing technologies?
    1. If so: what kinds of support in particular?
    2. Would your clinic be able to provide this support?

**Section 2: Clinic Culture**

1. Organisational culture
2. How would you characterise the workplace culture in this clinic?
   1. Would you describe it as an extended family with lots of personal sharing, or as a place where the main focus is getting the job done?
   2. Does the clinic have lots of rules and guidelines, or is it more like a dynamic company with room for innovation?
3. Multi-disciplinary working and team climate
4. Can you describe the management system at the clinic?
5. Are there any challenges in terms of professions being managed by other professions?
6. Is there a clear vision shared by the team, or different visions for different professions? How does this affect teamwork?
7. Can you describe how information sharing works in the clinic?
8. Resources
9. Do you feel the clinic has sufficient resources to carry out its work, in terms of (e.g.) staffing, training, and funding for treatments?
10. Are there particular challenges in your geographical area?
11. Are there differences in resources for different professions within the clinic?
12. Do the clinic systems (e.g. IT) support the work of the clinic or are there challenges in this regard?

**Table S1. Thematic Analysis Coding Framework**

| Name of code | Definition of code |  | Number of  participants | | Number of references | |
| --- | --- | --- | --- | --- | --- | --- |
| **1. Access pathway** | Way in which users gain access to technology |  | |  | |  |
| Attrition | Users who drop out of access pathway | 21 | | 26 | |  |
| Equity | Clinician attempts to ensure equitable access to technology | 29 | | 39 | |  |
| Format | Organisational characteristics of access pathway | 31 | | 107 | |  |
| Funding | National Health Service (NHS) arrangements for funding of technology access | 24 | | 51 | |  |
| NICE guidance | National Institute for Health and Care Excellence (NICE) guidance for technology access | 26 | | 58 | |  |
| *Definitions* | Terminology used in NICE guidance | 10 | | 13 | |  |
| *Flexibility* | Extent to which NICE terminology is open to interpretation | 22 | | 30 | |  |
| *Gaming* | Attempts by clinicians and users to manipulate NICE guidance | 9 | | 15 | |  |
| *Precision* | Extent to which NICE terminology encompasses objective criteria | 14 | | 25 | |  |
| Self-funding | Purchase of technology by users | 21 | | 25 | |  |
| Stakeholders | References to the range of actors involved in securing technology access | 10 | | 36 | |  |
| **2. Clinician characteristics** | Characteristics of individual clinician interviewees |  | |  | |  |
| Profession | Professional background | 36 | | 58 | |  |
| Type 1 contact | Degree to which clinicians come into contact with users with type 1 diabetes | 22 | | 23 | |  |
| Tech contact | Extent to which professional role requires involvement with technology | 33 | | 62 | |  |
| Tech opinions | Opinions regarding technology in general (not diabetes technology) | 33 | | 41 | |  |
| **3. Clinics** | Characteristics of participating outpatient hospital clinics |  | |  | |  |
| Clinic population | Characteristics of type 1 users served by the clinic | 20 | | 45 | |  |
| Information sharing | Formal and/or informal arrangements for information sharing within the clinic | 15 | | 16 | |  |
| Information technology | Level of information technology equipment and usage in the clinic | 32 | | 38 | |  |
| Levels of tech usage | Extent and kinds of technology use by type 1 users in the clinic population | 34 | | 62 | |  |
| Local challenges | Specific burdens faced by members of clinic population and treating clinicians | 5 | | 7 | |  |
| Management and vision | Degree and character of leadership at the clinic | 22 | | 26 | |  |
| Organisation | Organisational structure of the clinic | 22 | | 30 | |  |
| Organisational culture | Organisational culture of the clinic | 33 | | 49 | |  |
| Professional collaboration | Extent of multi-disciplinary working | 26 | | 43 | |  |
| Resources | Funding available to the clinic | 31 | | 47 | |  |
| Technology culture | Prevailing attitudes towards technology in the clinic | 26 | | 65 | |  |
| **4. Closed-loop** | Clinician attitudes towards and experience of closed-loop systems |  | |  | |  |
| Changes needed for care | Organisational and clinical transformations required to support future closed-loop usage in mainstream care | 28 | | 38 | |  |
| Closed-loop candidacy | Attributes regarded by clinicians as qualifying users for closed-loop access | 30 | | 41 | |  |
| Do-it-yourself closed-loop | Clinician attitudes towards users constructing and/or using self-made closed-loop systems | 2 | | 4 | |  |
| Envisaged benefit | Clinician expectation of benefits arising for future users of closed-loop systems | 20 | | 27 | |  |
| Experience of trials | Clinician experience of previous or current trials involving closed-loop technology | 10 | | 11 | |  |
| Knowledge of closed-loop | Degree of awareness regarding closed-loop technology | 18 | | 19 | |  |
| User limitations | Opinions regarding the impact of human factors on closed-loop usage | 22 | | 34 | |  |
| **5. Views on users** | Clinician opinions regarding users of currently available technologies such as insulin pumps and continuous glucose monitors |  | |  | |  |
| Adaptability | Extent to which users are able to modify technology use based on clinical advice | 26 | | 40 | |  |
| Brand differences | Extent to which user experience varies by technology brand | 17 | | 19 | |  |
| Candidacy | Attributes regarded by clinicians as qualifying users for closed-loop access | 32 | | 165 | |  |
| Expectations | Users’ expectations regarding contribution of technology to diabetes self-care | 17 | | 24 | |  |
| Predictability | Extent to which user experience and benefit can be predicted by clinicians | 35 | | 63 | |  |
| Proactivity | Extent to which users exhibit self-driven engagement with technology and technology access | 32 | | 80 | |  |
